# Editorial: Bispecific Antibodies for T-Cell Based Immunotherapy

**DOI:** 10.3389/fonc.2020.628005

**Published:** 2020-12-09

**Authors:** Brian H. Santich, Nai-Kong V. Cheung, Christian Klein

**Affiliations:** ^1^ Department of Pediatrics, Memorial Sloan Kettering Cancer Center, New York, NY, United States; ^2^ Cancer Immunotherapy Discovery, Roche Innovation Center Zurich, Schlieren, Switzerland

**Keywords:** bispecific antibody, T-cell, immunotherapy, protein engineering, oncology

## Bispecific Antibody Designs

To date, the FDA and EMA have approved bispecific antibodies (BsAb) using two different designs: the tandem-single-chain variable fragment (scFv) [blinatumomab (1)], and the heterodimeric IgG-molecule [emicizumab (2)]. However, more than 100 BsAb formats have been described in the literature, with varying molecular shapes, sizes, and valencies (3). While developmental considerations will always be an important decision, ultimately it is the functional properties of the design which dictate efficacy and safety. Vafa and Trinklein provide a valuable discussion of this subject, especially as it relates to epitopes for T-cell engagement. Of particular interest is the concept of decoupling cytokine release from anti-tumor cytotoxicity, thereby limiting the impact of cytokine release syndrome (CRS) and potentially permitting substantial increases in the maximum tolerated dose (MTD). Given the number of clinical trials which report CRS as the dose-limiting toxicity (4), such approaches, if confirmed in the clinic, could provide significant clinical benefit, and may also improve other CRS-inducing immunotherapies such as CAR-T cell therapy. Complementing this approach, Lum et al. have developed an alternative way to administer BsAbs by premixing or “arming” T-cells with BsAb ex-vivo prior to administration. This substantially reduces the total administrated BsAb dose, while still providing potent anti-tumor activity, as demonstrated in Dr. Lum’s recent work targeting CS-1. Whether these approaches will succeed in a clinical setting remains to be seen.

Alternatively, work from De Luca et al. exemplifies how T-cell engaging BsAbs can be designed without CD3 targeting. Instead, De Luca and colleagues designed a trimeric format that localized IL-2 and TNF to CAIX-expressing tumors, with the TNF cytokine used both as an immune cell agonist and a multimerization tag for the protein itself. Doing so allowed them to take advantage of a greater avidity when binding to immune cells (trimeric vs monomeric) without increasing the protein complexity through additional multimerization domains or higher affinity interactions.

It is not currently clear how many more antibody designs will eventually receive clinical approval; however, as we learn more about protein design and engineering, newer and more advanced formats will become available, and hopefully improve the bispecific antibody landscape at large. However, as long as safety and potency remain the most important endpoints, future optimizations should remain focused on cytokine release, T-cell activation and cytotoxicity.

## Treatments for B-Cell Malignancies: Myeloma and Lymphomas

B-cell malignancies remain one of the model diseases for T-cell BsAb or adoptive T-cell immunotherapies, with the only FDA/EMA approvals so far occurring in leukemias and lymphomas. Due to the highly lineage restricted protein expression of many of B-cell tumor antigens (CD19, CD20, CD22, etc) as well as the treatable side effects of short and long-term B cell aplasia, therapeutics directed at B-cell malignancies have generally seen more clinical successes than those against solid tumors. However, as reviewed by both Lejeune et al. and Caraccio et al., the presence of safe and specific tumor antigens has not made the treatment of these diseases simple, particularly for multiple myelomas (MM) and indolent Non-Hodgkin lymphomas (NHL), which both remain largely incurable for most patients.

Multiple myelomas have offered several targets for T-cell BsAb treatment, such as CD38, CD138 or BCMA. Among these, BCMA is generally considered the most promising, due to its relative absence on non-lymphoid tissues, stem cells, or T-cells, and has recently seen the approval of an antibody-drug conjugate (belantamab mafodotin-blmf). Consequently, the majority of ongoing clinical trials for the treatment of MM have focused on BCMA, as reviewed by Dr. Caraccio. Non-Hodgkin lymphomas typically express common B-cell antigens, such as CD19 and CD20, two targets with approved antibody (CD19: tafasitamab; CD20: rituximab/obinutuzumab, ofatumumab) or BsAb therapeutics (CD19: blinatumomab). Like MM, ongoing clinical trials are exploring a multitude of BsAb formats, and some groups are even exploring combining therapies with other modalities, such as immune checkpoint inhibition (ICI), immunomodulatory imide drugs (ImIds) or antibody-drug conjugates (ADCs).

Given the difficulty in treating solid tumors, B-cell malignancies such as MM and NHL, may be the next indications where BsAb therapies provide significant clinical impact. With the enormous diversity of antibody formats being tested and CD3 epitopes being targeted, once phase I trials are completed it will be interesting to compare their safety profiles in addition to their relative anti-tumor effect.

## Combination Treatments

Many groups have also begun studying combination therapies as an alternative method to overcome the limits of BsAb monotherapy, seeking to both improve the treatment efficacy and response duration. As demonstrated by Sam et al. combining T-cell engaging BsAbs with checkpoint blockade therapy, especially anti-PD-L1 immune checkpoint inhibitors, remains one of the most promising combinations to date. In addition to both PD-1 and PD-L1 being upregulated after treatment with BsAbs, both targets have multiple approved antibodies, greatly simplifying the clinical strategy for combination studies. Additionally, both T-cell BsAbs and ICI therapies have suffered from separate but potentially complementary limitations. T-cell BsAbs have struggled to treat solid tumors, while ICI therapies have had major successes treating lung cancer, colon cancer and melanoma. By contrast, ICI therapies are thought to require some kind of pre-existing immune infiltration, PD-L1 upregulation or T-cell immunity, with little efficacy in patients with so called “cold” or non-inflamed tumors (5), while T-cell BsAbs appear able to provide each of these functions quite effectively preclinically (6). It is therefore imaginable that T-cell BsAb therapy could be used to inflame an otherwise “cold” tumor and sensitize it to ICI therapy. It should be noted that with any synergy in efficacy comes the risk of synergy in toxicity, however this should be resolvable through appropriate dosing and treatment scheduling. Which targets and tumor types are most sensitive to such a combination therapy remains to be seen, however, judging by the study from Sam et al., MSI colon cancer appears very promising.

In summary, BsAb studies over the last several years have demonstrated compelling preclinical and early phase clinical data. This Research Topic explored many of these concepts and also proposed several new strategies for treating cancer with BsAbs. Going forward we hope to see each of these advance our understanding of T-cell immunotherapy and hope that some provide a much-needed improvement in clinical outcomes.

## Author Contributions

All authors listed have made a substantial, direct, and intellectual contribution to the work and approved it for publication.

## Conflict of Interest

BS and N-KC have been listed as inventors on patents filed by MSKCC. N-KC has received sponsored research grants from Y-mAbs, has financial interest in Y-mAbs, Abpro-Labs, and Eureka Therapeutics, and is a SAB member for Abpro-Labs and Eureka Therapeutics. CK declares employment, patents, and stock ownership with Roche.
